# Machine learning predicts the prognosis of breast cancer patients with initial bone metastases

**DOI:** 10.3389/fpubh.2022.1003976

**Published:** 2022-09-26

**Authors:** Chaofan Li, Mengjie Liu, Jia Li, Weiwei Wang, Cong Feng, Yifan Cai, Fei Wu, Xixi Zhao, Chong Du, Yinbin Zhang, Yusheng Wang, Shuqun Zhang, Jingkun Qu

**Affiliations:** ^1^Department of Oncology, The Second Affiliated Hospital of Xi'an Jiaotong University, Xi'an, China; ^2^Department of Radiation Oncology, The Second Affiliated Hospital of Xi'an Jiaotong University, Xi'an, China; ^3^Department of Otolaryngology, The Second Affiliated Hospital of Xi'an Jiaotong University, Xi'an, China

**Keywords:** breast cancer, bone metastases, XGBoost algorithm, neoadjuvant chemotherapy, SEER

## Abstract

**Background:**

Bone is the most common metastatic site of patients with advanced breast cancer and the survival time is their primary concern; however, we lack accurate predictive models in clinical practice. In addition to this, primary surgery for breast cancer patients with bone metastases is still controversial.

**Method:**

The data used for analysis in this study were obtained from the SEER database (2010–2019). We made a COX regression analysis to identify prognostic factors of patients with bone metastatic breast cancer (BMBC). Through cross-validation, we constructed an XGBoost model to predicting survival in patients with BMBC. We also investigated the prognosis of patients treated with neoadjuvant chemotherapy plus surgical and chemotherapy alone using propensity score matching and K–M survival analysis.

**Results:**

Our validation results showed that the model has high sensitivity, specificity, and correctness, and it is the most accurate one to predict the survival of patients with BMBC (1-year AUC = 0.818, 3-year AUC = 0.798, and 5-year survival AUC = 0.791). The sensitivity of the 1-year model was higher (0.79), while the specificity of the 5-year model was higher (0.86). Interestingly, we found that if the time from diagnosis to therapy was ≥1 month, patients with BMBC had even better survival than those who started treatment immediately (HR = 0.920, 95%CI 0.869–0.974, *P* < 0.01). The BMBC patients with an income of more than USD$70,000 had better OS (HR = 0.814, 95%CI 0.745–0.890, P < 0.001) and BCSS (HR = 0.808 95%CI 0.735–0.889, *P* < 0.001) than who with income of < USD$50,000. We also found that compared with chemotherapy alone, neoadjuvant chemotherapy plus surgical treatment significantly improved OS and BCSS in all molecular subtypes of patients with BMBC, while only the patients with bone metastases only, bone and liver metastases, bone and lung metastases could benefit from neoadjuvant chemotherapy plus surgical treatment.

**Conclusion:**

We constructed an AI model to provide a quantitative method to predict the survival of patients with BMBC, and our validation results indicate that this model should be highly reproducible in a similar patient population. We also identified potential prognostic factors for patients with BMBC and suggested that primary surgery followed by neoadjuvant chemotherapy might increase survival in a selected subgroup of patients.

## Introduction

Breast cancer (BC) now is the first most diagnosed cancer (11.7% of the new cancer cases) worldwide, accounts for a quarter of all female cancer cases and BC is also the leading cause of cancer death among female patients (1). With significant treatment advances, the survival of patients with BC was improved dramatically. However, distant metastases remain the leading cause of death in patients with BC (2) and a major challenge for clinicians.

Overall, the average proportion of all breast cancer patients with an initial diagnosis of bone metastases is about 5% (3), and bone metastases usually lead to skeletal-related events, namely, pain, pathological fractures, spinal cord compression, hypercalcemia, and other complications (4). Current treatments for bone metastases are limited and merely palliative; standard anti-osteoporotic agents, chemotherapy, and radiotherapy can delay or lessen skeletal-related events, but they cannot cure bone metastases (5), the 5-year overall survival rate is only 22.8% (6). Thus, the survival time is the most important concern for patients with bone metastatic breast cancer (BMBC) and clinicians. However, there is no accurate prediction model for them. The most used model for predicting the survival rate is nomogram, but its accuracy rate is only about 70% (7–12). As a result, a more accurate and powerful model is needed.

Nowadays, machine learning methods can create an artificial intelligence (AI) model to predict the survival of patients with cancer, which significantly increases the accuracy rate (13). However, machine learning algorithms also have drawbacks and need to be improved in practice. For example, a support-vector machine (SVM) is not good at handling large numbers of samples and variables, K-nearest neighbor (KNN) is not very interpretable, and decision trees are easy to train quickly, but not complex enough (14, 15). Whereas extreme gradient boosting (XGBoost) is created iteratively to minimize the loss function, which makes it perform well in many areas (16–18). But it is rarely applied in clinical patient prognosis prediction. Through inter-model comparison, we found that XGBoost also performed well on such prognostic problems.

This study examined the prognosis of patients with BMBC from the Surveillance Epidemiology and End Results (SEER) database. And we created a high-precision AI model to predict the 1-, 3-, and 5-year survival of patients with BMBC. This work provides insight into the factors that influence the prognosis of patients with BMBC and contributes to the development of a clinical model to improve the long-term follow-up of patients with BMBC.

## Materials and methods

### Data source and study design

**Figure 1:**
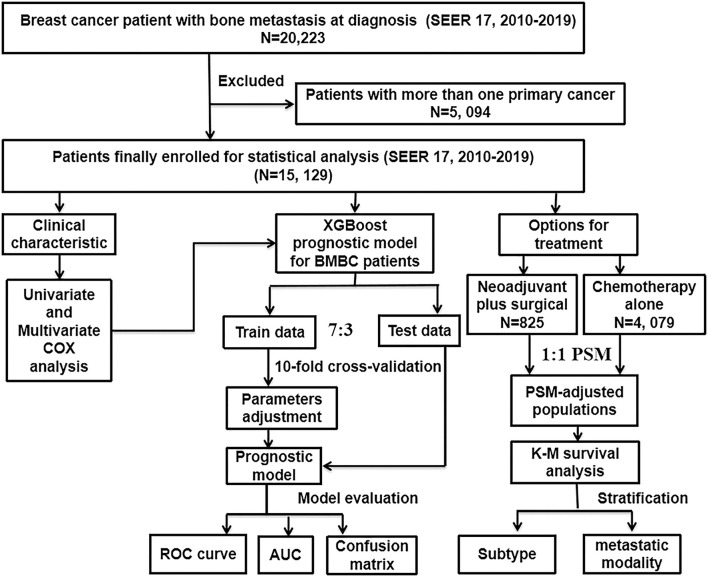
The flowchart described the process of conducting the study and statistical analysis. SEER, the Surveillance, Epidemiology, and End Results database; BMBC, bone metastatic breast cancer; PSM, propensity score matching; COX, concordance index; ROC curve, receiver operating characteristic curve; AUC, area under the curve; K–M, Kaplan–Meier; XGBoost, extreme gradient boosting.

The workflow of our study design and analyses is shown in Figure 1. As the information on distant metastases was included from 2010, the data used for analysis in this study were obtained from the Surveillance, Epidemiology, and End Results (SEER) database [SEER 17 Regs study data (changes 2010–2019); version 8.4.0]. Because the data are publicly available and do not include personally identifiable patient information, this retrospective cohort study was approved by the Institutional Review Board of the Second Affiliated Hospital of Xi'an Jiaotong University, which decided to waive informed consent. From this database, data were collected on women with BC. Inclusion criteria were as follows: (1) BC was only cancer diagnosed in the patient; (2) all patients with cancer had evidence of the International Classification of Cancer Diseases Edition III (ICD-O-3) morphological and histopathology diagnosis; (3) patient had bone metastases at initial diagnosis. Exclusion criteria was that patients were diagnosed with more than one primary cancer. In this study, patients were followed up until death, loss to follow-up or December 31, 2019.

### Statistical analysis

We did univariate COX regression models to analyze the relationship between various clinical and pathological characteristics and patient survival. Further multifactorial COX analysis was done to compare the risk of death of patients and to identify independent factors of prognosis. To investigate the impact of neoadjuvant chemotherapy plus surgical treatment on the prognosis of patients with BMBC, patients receiving neoadjuvant chemotherapy plus surgical and chemotherapy alone, respectively, were matched on a 1:1 propensity score (PSM) based on statistically significant variables in multifactorial COX analysis. A Kaplan–Meier (K–M) survival analysis stratified by metastatic modality and molecular subtype was also performed on the PSM-adjusted population. All statistical analyses were performed using R software (version 4.0.2). A bilateral tail value of < 0.05 was considered statistically significant.

### XGBoost model

XGBoost is a modification of the gradient boosting algorithm, using Newton's method when solving for the extreme values of the loss function, Taylor expansion of the loss function to the second order, and additionally a regularization term is added to the loss function. The objective function at training time consists of two parts, the first part being the gradient boosting algorithm loss and the second part being the regularization term. The principle of the XGBoost algorithm can be summarized as follows: feature vector with the corresponding (output) category **yi:**


(1) $$\begin{array}{l} yi\text{Ĺ}=\sum k=1Kfk\left(xi\right),fk\in F, \end{array}$$

Feature selection: univariate and multivariate COX analyses were performed on the clinical characteristics extracted from the SEER database, and statistically significant characteristics, namely, age at diagnosis, race, marital status, months from diagnosis to therapy, T stage, N stage, grade, breast subtype, median household income, distant sites of metastases, and treatment information were incorporated into the machine learning model to predict 1-, 3-, and 5-year overall survival for patients with BMBC. These analyses were performed before the exclusion of patients who were alive but survived less than 1, 3, or 5 years at the follow-up cut-off date. Before running the training program, a response variable was obtained for survival information, in which 1 = survival and 0 = death. Patients were randomly divided into train data and test data according to 7:3. And we compared the performance of SVM, decision tree (ID3), K-Nearest Neighbor (KNN), and extreme gradient boosting (XGBoost) on test data. Receiver operating characteristic (ROC) analysis, area under the ROC curve (AUC), and confusion matrix were used for the evaluation of the model. The main evaluation indicators in the confusion matrix include sensitivity, specificity, and correctness.

## Results

### Clinical characteristics of patients with BMBC

Ultimately, we extracted the information of 15,129 eligible patients with BMBC in the SEER database from 2010 to 2019. The clinic-pathological characteristics of BC patients with bone metastases are shown in Table 1 and summarized as follows. The mean age of patients was 61.39 years, with 1,113 (7.36%) patients younger than 40 and 1,752 (11.58%) patients older than 80. Five thousand six hundred nine (37.07%) patients received treatment immediately after diagnosis, while 7,271 (48.06%) patients received treatment more than 1 month after diagnosis. The molecular subtype of HR+/HER2– accounted for 57.19%, followed by HR+/HER2+ (14.22%) and HR–/HER2– (7.86%), and HR–/HER2+ only accounted for 6.05%. In terms of race, 76.70% of the patients were white. The most common histological type was infiltrating ductal carcinoma (IDC) (63.68%). In terms of marital status, 42.44% of the patients were married and 23.46% were single. The staging T1–T4 was 11.78, 28.72, 14.93, and 29.49%; and the percentages of N0–N3 were 21.13, 43.75, 9.72 and 20.26%, respectively. About 30.98% of the patients had a grade III or IV tumor grade pathology, while only 6.70% had a grade I. About 38.35% of the patients had a good family financial situation with an annual income of more than US$70,000. In terms of treatment, 20.37% of patients received surgical treatment, 34.15% received radiotherapy, 53.06% received chemotherapy, and 7% received neoadjuvant chemotherapy. Brain metastases, liver metastases, lung metastases, distant lymph nodes, and other distant organs metastases accounted for 7.31, 24.39, 26.75, 9.83, and 6.46% of patients, respectively.

**Table 1:** Baseline characteristics of patients with bone metastatic breast cancer (BMBC) included from SEER data cohort.

**Characteristic**		**Cases**	**%**
Age at diagnosis	< 40	1,113	7.36
	40–49	1,962	12.97
	50–59	3,674	24.28
	60–69	3,985	26.34
	70–79	2,643	17.47
	80+	1,752	11.58
	0 month	5,609	37.07
Months from diagnosis to therapy	≥1 month	7,271	48.06
	unknown	2,249	14.87
	HR+/HER2–	8,652	57.19
	HR+/HER2+	2,151	14.22
Subtype	HR–/HER2+	915	6.05
	HR–/HER2–	1,189	7.86
	Unknown	2,222	14.69
Race	White	11,604	76.70
	Black	2,042	13.50
	Other	1,483	9.80
Histological type	IDC	9,634	63.68
	ILC	1,710	11.30
	Mixed	840	5.55
	Other	2,945	19.47
	Married	6,421	42.44
Marriage status	Singled	3,550	23.46
	Widow/divorced/other	5,158	34.09
T Stage	T1	1,782	11.78
	T2	4,345	28.72
	T3	2,259	14.93
	T4	4,461	29.49
	Unknown	2,282	15.08
N Stage	N0	3,197	21.13
	N1	6,619	43.75
	N2	1,471	9.72
	N3	3,065	20.26
	Unknown	777	5.14
Grade	I; well differentiated	1,014	6.70
	II; moderate differentiated	5,115	33.81
	III/IV; poorly differentiated	4,687	30.98
	Unknown	4,313	28.51
Median household income (inflation adjusted)	< 50,000$	1,881	12.43
	50,000–59,999$	2,416	15.97
	60,000–69,999$	5,030	33.25
	70,000$	5,802	38.35
Neoadjuvant chemotherapy	No/unknown	14,097	93.18
	Yes	1,032	6.82
Chemotherapy	No/unknown	7,102	46.94
	Yes	8,027	53.06
Radiotherapy	No/unknown	9,962	65.85
	Yes	5,167	34.15
Surgery	No/unknown	12,047	79.63
	Yes	3,082	20.37
Liver metastases	No/unknown	11,439	75.61
	Yes	3,690	24.39
Lung metastases	No/unknown	11,082	73.25
	Yes	4,047	26.75
Brain metastases	No/unknown	14,023	92.69
	Yes	1,106	7.31
Distant lymph nodes metastases	No/unknown	13,642	90.17
	Yes	1,487	9.83
Distant other metastases	No/unknown	14,151	93.54
	Yes	978	6.46

### Univariable and multivariable COX regression analyses

We performed univariate COX regression to identify significant variables affecting overall survival (OS) and breast cancer-specific survival (BCSS) in patients with BMBC, namely, age at diagnosis, months from diagnosis to therapy, molecular subtype, race, histological type, marital status, T stage, N stage, grade, household income (inflation-adjusted), treatment, and distant metastases information (Table 2).

**Table 2:** Univariate and multivariate COX analyses of characteristics extracted from the SEER database.

	**Univariate COX analysis**						**Multivariate COX analysis**					
	**OS**			**BCSS**			**OS**			**BCSS**		
	**HR**	**95%CI**	***P* value**	**HR**	**95%CI**	***P* value**	**HR**	**95%CI**	***P* value**	**HR**	**95%CI**	***P* value**
**Age at diagnosis**												
< 40	Reference			Reference			Reference			Reference		
40–49	1.053	0.950–1.166	0.325	1.067	0.958–1.189	0.236	0.999	0.879–1.134	0.983	0.997	0.873–1.139	0.964
50–59	1.407	1.284–1.543	***	1.398	1.268–1.541	***	1.198	1.067–1.344	**	1.180	1.045–1.331	**
60–69	1.583	1.446–1.734	***	1.545	1.403–1.701	***	1.370	1.218–1.541	***	1.332	1.177–1.507	***
70–79	1.932	1.758–2.123	***	1.811	1.639–2.003	***	1.517	1.334–1.724	***	1.444	1.261–1.653	***
80+	2.908	2.637–3.206	***	2.598	2.440–2.886	***	2.250	1.951–2.596	***	2.028	1.741–2.363	***
**Months from diagnosis to therapy**												
0 month	Reference			Reference			Reference			Reference		
≥1 month	0.850	0.813–0.889	***	0.840	0.801–0.881	***	0.920	0.869–0.974	**	0.909	0.856–0.966	**
**Subtype**												
HR+/HER2–	Reference			Reference			Reference			Reference		
HR+/HER2+	0.796	0.747–0.848	***	0.809	0.756–0.866	***	0.725	0.665–0.790	***	0.713	0.651–0.781	***
HR–/HER2+	1.082	0.991–1.181	0.079	1.100	1.002–1.208	*	0.957	0.849–1.078	0.468	0.923	0.813–1.048	0.216
HR–/HER2-	2.593	2.422–2.776	***	2.761	2.567–2.971	***	2.801	2.548–3.078	***	2.870	2.600–3.169	***
**Race**												
White	Reference			Reference			Reference			Reference		
Black	1.310	1.239–1.386	***	1.288	1.212–1.368	***	1.320	1.217–1.431	***	1.290	1.183–1.407	***
Other	0.923	0.859–0.991	*	0.921	0.853–0.995	*	1.013	0.916–1.119	0.806	0.990	0.890–1.102	0.858
**Histological type**												
IDC	Reference			Reference			Reference			Reference		
ILC	1.048	0.983–1.118	0.152	1.032	0.963–1.106	0.367	1.249	1.135–1.374	***	1.251	1.129–1.385	***
Mixed	0.894	0.818–0.977	*	0.901	0.821–0.990	*	1.055	0.946–1.176	0.339	1.069	0.953–1.201	0.256
Other	1.763	1.680–1.850	***	1.646	1.562–1.735	***	1.267	1.134–1.416	***	1.243	1.104–1.399	***
**Marriage status**												
Married	Reference			Reference			Reference			Reference		
Singled	1.256	1.193–1.323	***	1.230	1.164–1.300	***	1.173	1.089–1.262	***	1.135	1.050–1.228	**
Widow/divorced/other	1.425	1.361–1.491	***	1.366	1.301–1.435	***	1.140	1.065–1.220	***	1.127	1.049–1.212	**
**T Stage**												
T1	Reference			Reference			Reference			Reference		
T2	1.025	0.954–1.102	0.496	1.041	0.963–1.125	0.312	1.049	0.949–1.160	0.352	1.030	0.926–1.147	0.583
T3	1.145	1.057–1.241	***	1.217	1.117–1.327	***	1.209	1.083–1.350	***	1.221	1.086–1.372	***
T4	1.424	1.328–1.528	***	1.487	1.378–1.604	***	1.244	1.124–1.378	***	1.235	1.108–1.377	***
**N stage**												
N0	Reference			Reference			Reference			Reference		
N1	0.907	0.861–0.956	***	0.942	0.890–0.997	*	1.003	0.927–1.085	0.934	1.017	0.935–1.106	0.697
N2	0.850	0.787–0.918	***	0.876	0.806–0.952	**	1.042	0.937–1.159	0.445	1.063	0.949–1.191	0.290
N3	1.099	1.036–1.166	**	1.124	1.054–1.198	***	1.142	1.041–1.252	**	1.162	1.053–1.282	**
**Grade**												
Well differentiated	Reference			Reference			Reference			Reference		
Moderately differentiated	1.172	1.068–1.287	***	1.189	1.076–1.314	***	1.304	1.165–1.460	***	1.333	1.180–1.506	***
Poorly differentiated	1.659	1.513–1.820	***	1.720	1.557–1.900	***	1.858	1.652–2.091	***	1.952	1.719–2.217	***
**Median household income(inflation adjusted)**												
< 50,000$	Reference			Reference			Reference			Reference		
50,000–59,999$	0.956	0.889–1.028	0.225	0.982	0.908–1.062	0.648	0.998	0.903–1.102	0.962	1.021	0.918–1.135	0.706
60,000–69,999$	0.914	0.857–0.974	**	0.931	0.869–0.997	*	0.907	0.830–0.992	*	0.918	0.835–1.009	0.077
70,000$	0.832	0.781–0.886	^***^	0.835	0.780–0.894	^***^	0.814	0.745–0.890	^***^	0.808	0.735–0.889	^***^
**Neoadjuvant chemotherapy**												
No/unknown	Reference			Reference			Reference			Reference		
Yes	0.454	0.413–0.499	***	0.472	0.427–0.522	***	0.802	0.714–0.900	***	0.805	0.712–0.909	***
**Chemotherapy**												
No/unknown	Reference			Reference			Reference			Reference		
Yes	0.550	0.529–0.572	***	0.585	0.561–0.611	***	0.672	0.629–0.718	***	0.685	0.639–0.735	***
**Radiotherapy**												
No/unknown	Reference			Reference			Reference			Reference		
Yes	0.783	0.751–0.817	***	0.801	0.766–0.839	***	1.098	1.035–1.165	**	1.101	1.034–1.173	**
**Surgery**												
No/unknown	Reference			Reference			Reference			Reference		
Yes	0.505	0.479–0.533	***	0.514	0.486–0.544	***	0.648	0.604–0.696	***	0.647	0.600–0.697	***
**Liver metastases**
No/unknown	Reference			Reference			Reference			Reference		
Yes	1.812	1.734–1.894	***	1.882	1.796–1.973	***	1.752	1.637–1.875	***	1.825	1.698–1.960	***
**Lung metastases**
No/unknown	Reference			Reference			Reference			Reference		
Yes	1.593	1.525–1.663	***	1.596	1.523–1.671	***	1.298	1.216–1.385	***	1.282	1.196–1.373	***
**Brain metastases**
No/unknown	Reference			Reference			Reference			Reference		
Yes	1.976	1.843–2.119	***	2.056	1.910–2.214	***	1.829	1.649–2.030	***	1.850	1.658–2.064	***
**Distant Lymph nodes metastases**
No/unknown	Reference			Reference			Reference			Reference		
Yes	1.246	1.155–1.345	***	1.244	1.146–1.350	***	1.002	0.888–1.130	0.980	1.004	0.883–1.140	0.955
**Distant other metastases**
No/unknown	Reference			Reference			Reference			Reference		
Yes	1.580	1.450–1.722	***	1.567	1.428–1.719	***	1.296	1.112–1.510	***	1.291	1.098–1.518	**

*P < 0.05, **P < 0.01, ***P < 0.001.

To identify independent variables associated with OS and BCSS, we then conducted multivariable COX regression (Table 2). We found that patients older than 50 years old, < 1 month from diagnosis to therapy, black race, ILC, T Stage>T3, Stage N3, moderately or high Grade, and visceral metastases (brain, liver, lung, or other) were significantly related to worse OS and BCSS. Compared with patients with HR+/HER2–, the HR+/HER2+ subtype revealed improved OS and BCSS, while HR–/HER2– subtype showed the worst outcome, and there was no difference between HR+/HER2– and HR–/HER2+. For treatment, primary tumor surgery, chemotherapy, and neoadjuvant chemotherapy could prolong OS and BCSS; however, radiotherapy showed the opposite effect in multivariable COX regression analysis. Some social factors like marital status and income situation were also associated with survival, married status, and annual household income of more than USD$70,000 were significantly related to better survival.

### Benefits of neoadjuvant chemotherapy plus surgical treatment in BMBC patients subdivided by molecular subtypes and metastatic sites

Neoadjuvant chemotherapy data were just opened by SEER; thus, we explored the role of this factor in the prognosis of patients with BMBC. We compared baseline characteristics between neoadjuvant chemotherapy plus surgical treatment and chemotherapy alone groups (Table 3). Patients in the neoadjuvant chemotherapy plus surgical group were younger, later T, N stages, and worse pathology grade, more likely to be married, hormone receptor negative and received surgery, and radiotherapy and chemotherapy. In addition, the neoadjuvant chemotherapy plus surgical group also included fewer liver, lung, brain and other distant metastases. Propensity score matching (PSM) was used to adjust for the observed imbalance. And no significant differences were seen in baseline characteristics after PSM adjustment (Table 3).

**Table 3:** Comparison of patient characteristics according to the use of neoadjuvant chemotherapy before and after propensity score matching (PSM).

**Characteristics**	**Unmatched cohort**					**1:1 propensity score matched (PSM) cohort**				
	**Neoadjuvant**+**surgical**		**Chemotherapy alone**		**Unadjusted**	**Neoadjuvant**+**surgical**		**Chemotherapy alone**		**PSM-adjusted**
	***N* = 825**	**%**	***N* = 4,079**	**%**	***P* value**	***N* = 715**	**%**	***N* = 715**	**%**	***P* value**
**Age at diagnosis**					< 0.001					0.562
< 40	152	18.42	368	9.02		112	15.66	122	17.06	
40–49	188	22.79	625	15.32		164	22.94	147	20.56	
50–59	236	28.61	1,186	29.08		207	28.95	213	29.79	
60–69	169	20.48	1,162	28.49		155	21.68	150	20.98	
70–79	67	8.12	553	13.56		64	8.95	61	8.53	
80+	13	1.58	185	4.54		13	1.82	22	3.08	
**Months from diagnosis to therapy**					0.001					0.601
0	296	35.88	1,724	42.27		266	37.20	269	37.62	
≥1	526	63.76	2,322	56.93		446	62.38	445	62.24	
Unknown	3	0.36	33	0.81		3	0.42	1	0.14	
**Subtype**					< 0.001					0.688
HR+/HER2–	431	52.24	2,229	54.65		380	53.15	365	51.05	
HR+/HER2+	196	23.76	740	18.14		169	23.64	161	22.52	
HR–/HER2+	79	9.58	368	9.02		63	8.81	71	9.93	
HR–/HER2–	95	11.52	402	9.86		80	11.19	95	13.29	
Unknown	24	2.91	340	8.34		23	3.22	23	3.22	
**Race**					0.137					0.161
White	619	75.03	2,998	73.50		538	75.24	525	73.43	
Black	107	12.97	635	15.57		94	13.15	118	16.50	
Other	99	12.00	446	10.93		83	11.61	72	10.07	
**Histological type**					< 0.001					0.827
IDC	666	80.73	2,760	67.66		576	80.56	586	81.96	
ILC	49	5.94	436	10.69		46	6.43	38	5.31	
Mixed	56	6.79	201	4.93		45	6.29	43	6.01	
Other	54	6.55	682	16.72		48	6.71	48	6.71	
**Marriage status**					0.053					0.769
Married	514	62.30	1,891	46.36		352	49.23	343	47.97	
Single	255	30.91	1,001	24.54		182	25.45	194	27.13	
Others	263	31.88	1,187	29.10		181	25.31	178	24.90	
**T stage**					< 0.001					0.958
T1	63	7.64	523	12.82		60	8.39	55	7.69	
T2	238	28.85	1,076	26.38		206	28.81	203	28.39	
T3	180	21.82	609	14.93		149	20.84	149	20.84	
T4	325	39.39	1,343	32.92		281	39.30	285	39.86	
Tx	19	2.30	528	12.94		19	2.66	23	3.22	
**N stage**					< 0.001					0.664
N0	94	11.39	882	21.62		91	12.73	78	10.91	
N1	377	45.70	1,984	48.64		344	48.11	339	47.41	
N2	163	19.76	302	7.40		121	16.92	123	17.20	
N3	187	22.67	736	18.04		155	21.68	168	23.50	
Nx	4	0.48	175	4.29		4	0.56	7	0.98	
**Grade**					< 0.001					0.885
Well	52	6.30	257	6.30		50	6.99	52	7.27	
Moderately	319	38.67	1,345	32.97		290	40.56	275	38.46	
Poorly	387	46.91	1,360	33.34		341	47.69	353	49.37	
unknown	67	8.12	1,117	27.38		84	11.75	85	11.89	
**Median household income (inflation adjusted)**					0.333					0.920
< 50,000$	94	11.39	483	11.84		77	10.77	77	10.77	
50,000–59,999$	142	17.21	634	15.54		122	17.06	124	17.34	
60,000–69,999$	286	34.67	1,345	32.97		254	35.52	242	33.85	
70,000+	303	36.73	1,617	39.64		262	36.64	272	38.04	
**Radiotherapy**					< 0.001					0.748
No/unknown	304	36.85	2,658	65.16		302	42.24	308	43.08	
Yes	521	63.15	1,421	34.84		413	57.76	407	56.92	
**Liver metastases**					< 0.001					0.883
No/unknown	713	86.42	2,776	68.06		606	84.76	604	84.48	
Yes	112	13.58	1,303	31.94		109	15.24	111	15.52	
**Lung metastases**					< 0.001					0.650
No/unknown	720	87.27	2,825	69.26		610	85.31	616	86.15	
Yes	105	12.73	1,254	30.74		105	14.69	99	13.85	
**Brain metastases**					< 0.001					0.429
No/unknown	814	98.67	3,699	90.68		704	98.46	700	97.90	
Yes	11	1.33	380	9.32		11	1.54	15	2.10	
**Distant other metastases**					< 0.001					0.759
No/unknown	802	97.21	3,724	91.30		694	97.06	692	96.78	
Yes	23	2.79	355	8.70		21	2.94	23	3.22	

**Figure 2:**
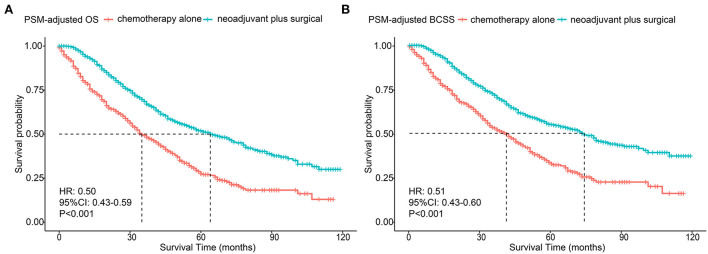
PSM-adjusted OS and BCSS of BMBC patients with neoadjuvant chemotherapy plus surgical treatment and chemotherapy alone. Kaplan–Meier (K–M) survival analysis: **(A)** OS of BMBC patients with neoadjuvant chemotherapy plus surgical treatment and chemotherapy alone; **(B)** BCSS of BMBC patients with neoadjuvant chemotherapy plus surgical treatment and chemotherapy alone. PSM, Propensity score matching OS, overall survival; BCSS, breast cancer-specific survival; BMBC, bone metastatic breast cancer; HR, hazard ratio; CI, confidence interval.

**Figure 3:**
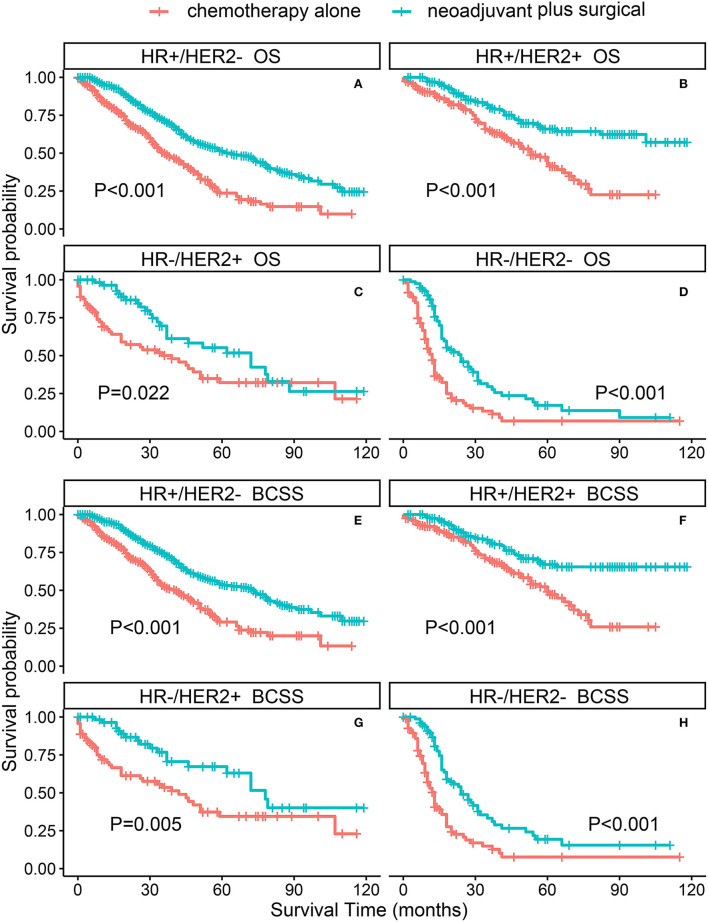
PSM-adjusted OS and BCSS of BMBC patients with neoadjuvant chemotherapy plus surgical treatment and chemotherapy alone (stratified by molecular subtype). Kaplan–Meier (K–M) survival analysis: **(A)** OS of BMBC patients with HR+/HER2- subtype; **(B)** OS of BMBC patients with HR+/HER2+ subtype; **(C)** OS of BMBC patients with HR–/HER2+ subtype; **(D)** OS of BMBC patients with HR–/HER2– subtype; **(E)** BCSS of BMBC patients with HR+/HER2– subtype; **(F)** BCSS of BMBC patients with HR+/HER2+ subtype; **(G)** BCSS of BMBC patients with HR–/HER2+ subtype; **(H)** BCSS of BMBC patients with HR-/HER2- subtype. OS, overall survival; BCSS, breast cancer-specific survival; BMBC, bone metastatic breast cancer; HR, hormone receptor; HER2, human epidermal growth factor receptor 2; PSM, propensity score matching.

**Figure 4:**
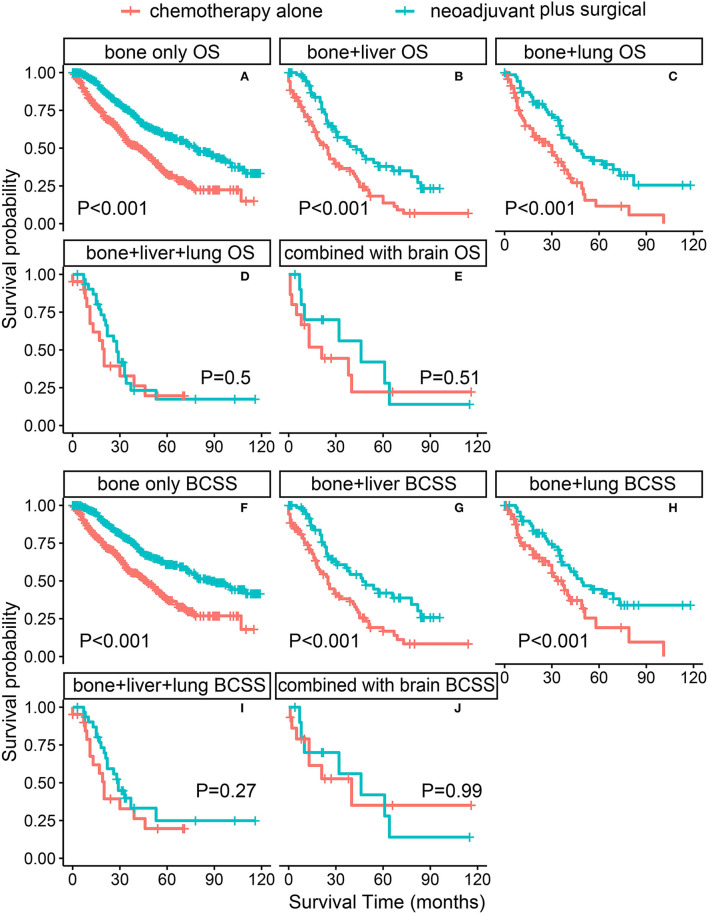
PSM-adjusted OS and BCSS of patients with BMBC in the neoadjuvant chemotherapy plus surgical treatment and chemotherapy alone groups (stratified by metastatic modality). Kaplan–Meier (K–M) survival analysis: **(A)** OS of patients with bone metastases only; **(B)** OS of patients with bone and liver metastases; **(C)** OS of patients with bone and lung metastases; **(D)** OS of patients with bone and liver and lung metastases; **(E)** OS of patients with BMBC combined with brain metastases; **(F)** BCSS of patients with bone metastases only; **(G)** BCSS of patients with bone and liver metastases; **(H)** BCSS of patients with bone and lung metastases; **(I)** BCSS of patients with bone and liver and lung metastases; **(J)** BCSS of patients with BMBC combined with brain metastases. OS, overall survival; BCSS, breast cancer-specific survival; PSM, Propensity score matching; BMBC, bone metastatic breast cancer.

The PSM-adjusted data showed about a 50% reduction in the overall risk of death in the neoadjuvant chemotherapy plus surgical group (*p* < 0.001, HR: 0.50; 95% CI: 0.43–0.59), which were similar to the results of multifactorial COX and allayed our concerns about selection bias in the PSM process (Figures 2A,B). Stratified K–M survival analysis showed that compared with chemotherapy alone, neoadjuvant chemotherapy plus surgical treatment significantly improved OS and BCSS in all molecular subtypes of patients with BMBC (Figures 3A–H; Supplementary Table 1). In addition to this, neoadjuvant chemotherapy plus surgical treatment significantly improved the OS and BCSS of patients with BMBC suffering from bone metastases only (Figures 4A,F), bone and liver metastases (Figures 4B,G), and bone and lung metastases (Figures 4C,H). In contrast, there was no significant difference in OS and BCSS of patients with BMBC who suffered from both liver and lung metastases (Figures 4D,I) or combined with brain metastases (Figures 4E,J; Supplementary Table 1).

### Establishing and evaluating predictive models for estimating the prognosis of patients with BMBC

**Figure 5:**
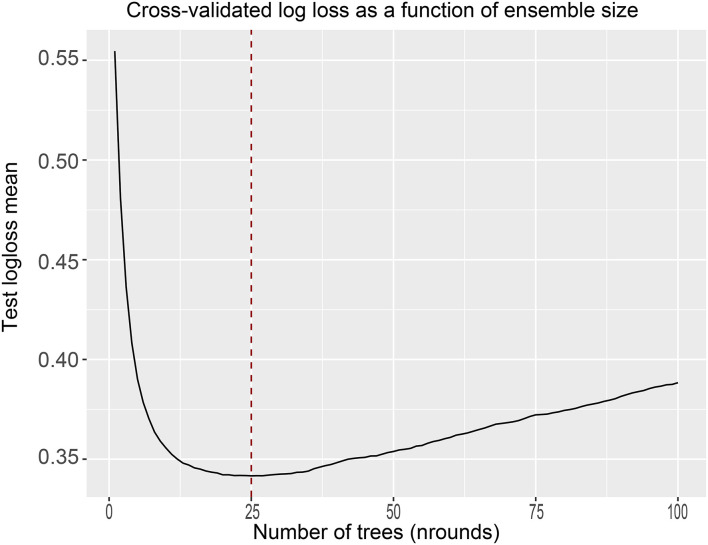
10-fold cross-validation in the train set to determine the optimal number of subtrees.

**Figure 6:**
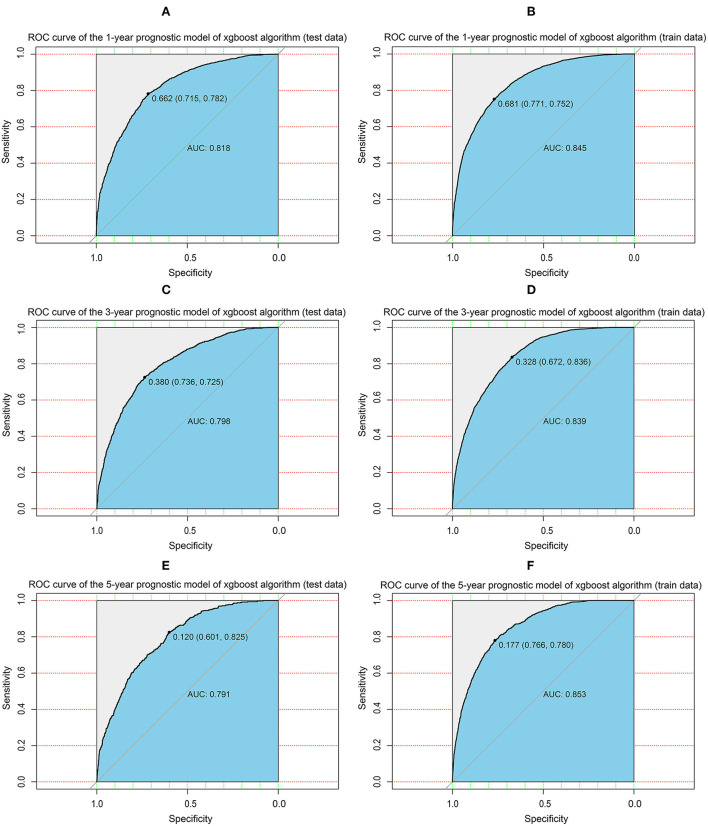
XGBoost model evaluation. **(A)** ROC curve for the 1-year prognostic model (test data); **(B)** ROC curve for the 1-year prognostic model (train data); **(C)** ROC curve for the 3-year prognostic model (test data); **(D)** ROC curve for the 3-year prognostic model (train data); **(E)** ROC curve for the 5-year prognostic model (test data); **(F)** ROC curve for the 5-year prognostic model (train data); ROC, receiver operating characteristic curve; AUC, area under the curve; XGBoost, extreme gradient boosting.

Given these results, we sought to build an XGBoost prediction model to estimate the OS of patients with BMBC at 1, 3, and 5 years. We divided the patients into train and test data according to 7:3, and to ensure the stability of the model, 10-fold cross-validation was used in the train set to assess the optimal number of subtrees. As shown in the figure, the logarithmic loss function was minimized at a number of 25 subtrees (Figure 5). From this, the “nrounds” parameter is determined and the model is then iteratively tested and adjusted to confirm other main hyperparameters to obtain the best model. We constructed predicted ROC curves for both the train and validation sets and calculated the corresponding AUCs. Our XGBoost model was highly effective in predicting the survival of patients with BMBC at 1 year (test set: AUC = 0.818; train set AUC = 0.845), 3 years (test set: AUC = 0.798; train set AUC = 0.839), and 5 years (test set: AUC = 0.791; train set AUC = 0.853) (Figure 6). Compared to traditional machine learning algorithms, SVM (1 year: AUC = 0.604; 3 years: AUC = 0.678; 5 years: AUC = 0.545), ID3 (1 year: AUC = 0.655; 3 years: AUC = 0.710; 5 years: AUC = 0.668) and KNN models (1 year: AUC = 0.607; 3 years: AUC = 0.664; 5 years: AUC = 0.596), XGBoost model performed significantly better (Table 4).

**Table 4:** Performance of prognostic models built by machine learning algorithms on the test data (area under the ROC curve).

	**1-year survival**	**3-year survival**	**5-year survival**
XGBoost	0.818	0.798	0.791
ID3	0.655	0.71	0.668
SVM	0.604	0.678	0.545
KNN	0.607	0.664	0.596

Then, the accuracy of our XGBoost model was further evaluated by the confusion matrix. The 1-year survival prediction model was calculated to have a sensitivity of 0.79, a specificity of 0.72, and a correctness of 0.77 (Supplementary Figure 1A); the 3-year survival model had a sensitivity of 0.66, a specificity of 0.76, and a correctness of 0.74 (Supplementary Figure 1B); and the 5-year survival model had a sensitivity of 0.57, a specificity of 0.86, and a correctness of 0.85 (Supplementary Figure 1C). The 1-year model seemed more sensitive and the 3 and 5-year models were more specific. Overall, our models performed well.

We also assessed the ranking of clinical characteristics in terms of importance in the model. The results showed that molecular subtype, surgical treatment, age, liver metastases, and chemotherapy were the top five determinants of patient survival. Among them, the molecular subtype is the most important factor. In addition, chemotherapy was an important factor for short-term survival (1 year) (Supplementary Figure 2A), while surgery was more important for medium- to long-term patient survival (3 and 5 years) (Supplementary Figures 2B,C).

## Discussion

Breast cancer exhibits metastatic properties, including bone, lung, liver, and brain, which leads to varied responses to treatment and patient prognosis (2). Bone metastases account for approximately 75% of metastatic cases (19), for these largest group patients of BMBC, who are considered incurable, the survival time is the most important concern. However, there is a lack of accurate prediction models in the clinic. Recently, some studies used nomograms to make several survival prediction models for patients with BMBC (7–12), but their accuracy rate is only about 70%. As a result, a more accurate and powerful model is needed. To our knowledge, the current study is the largest one to analyze the clinical characteristics and prognosis of patients with BMBC. The 1-, 3-, and 5-year OS of patients with BMBC is 66.39, 34.78 and 15.28%, respectively. Moreover, this study is the first to create an AI prognostic model for patients with BMBC and the model we made is the most accurate one to predict the survival of patients with BMBC.

In this analysis, several factors associated with improved outcomes were identified, including age < 50 years old, HR+/HER2+ subtype, white race, lower grade, lower T stage (T ≤ T2), no concurrent visceral metastases, ≥ 1 month from diagnosis to therapy, married, and income more than USD$70,000. Previous studies showed patients with BMBC of age < 40 years old were prone to better OS (6), while another one indicated the age < 60 years old was a protective factor (11), we analyzed more age groups, and found age < 50 years old was a feature for better OS and BCSS and the HR was increased with older age. The patients of the HR+/HER2+ subtype, rather than HR+/HER2– which usually present a preferred prognosis, showed the best survival among all subtypes in our analysis. This finding was similar to several previous studies (6, 11), and it might attribute to the progress of HER2 targeted therapy. Interestingly, we found if the time from diagnosis to treatment start was more than 1 month, the survival of patients with BMBC was even better than those with immediate treatment start. Of course, this does not mean the later treatment is better, if patients extended treatment delay indefinitely, they would die sooner. Treatment delays have a measurable impact on outcomes. Optimal times from diagnosis are < 90 days for surgery, < 120 days for chemotherapy, and < 365 days for radiotherapy (20). In large-scale hospitals, there can be more choices of systemic treatment, and the possible selection of optimal clinical trials can also have benefits for later-stage patients. Moreover, some previous studies have reported that shorter treatment delays are associated with poorer survival, because urgent treatment may be preferentially offered to patients who exhibit a higher symptom burden, which might lead to a worse prognosis (21–24). In addition, the treatment options also affect the time from diagnosis to treatment (25), for example, patients receiving chemotherapy alone or forgoing systemic therapy may have a shorter time from diagnosis to treatment. Our results imply that patients should not be worried about needing immediate treatment after diagnosis with BMBC, waiting for some relevant genetic or laboratory test to assess comprehensively their condition, and even searching for some appropriate clinical trials to enroll in could improve the therapeutic effect and prolong the survival time. It is reported that family income could affect the survival of patients with breast cancer (26); usually, patients with higher incomes have a better prognosis. We found that BMBC patients with an income of more than USD$70,000 had better OS and BCSS than those with income < USD$50,000, this income level dividing line was not reported before in this population, which could reflect their degree of cooperation with doctors in treatment.

For treatment, we found that primary tumor surgery, chemotherapy, and neoadjuvant chemotherapy could prolong OS and BCSS of patients with BMBC; however, radiotherapy showed an opposite effect in our analysis. Some studies showed that radiotherapy was not an independent prognostic factor of patients with BMBC (8, 10, 12), while other analyses reported that radiotherapy was associated with a significant survival advantage in patients with *de novo* Stage IV Breast Cancer (27, 28). These contradictory results may be due to different populations and clinical characteristics; we still need more detailed analysis to identify what kinds of patients would benefit from radiotherapy. Another controversial topic is whether surgical therapy for the primary site improves survival in patients presenting with *de novo* metastatic breast cancer. Many retrospective analyses of large cohort or mono-centric databases have shown a better prognosis of primary surgery in selected patients (11, 29–34); however, several randomized controlled trials indicated conflicting evidence (35–37), and a multicenter Turkish trial MF07-01 showed no difference in surgery arm of 3-year follow-up, but a statistically significant improvement in surgery arm of 4- to 10-year follow-up (38). We know that retrospective results are usually undermined for selection bias (women receiving surgery were younger and had biologically favorable tumors) (37), while prospective trials were also questioned for insufficient chemotherapy, deviation from contemporary practice, insufficient adapted *p*-value, and so on (11). Although each method has limitations and shows contradictory results, current studies imply that in well-selected patients, primary surgery might be a treatment option.

In this study, due to the SEER data of neoadjuvant chemotherapy therapy being first to open in April 2022, we are the first one to analyze the survival of patients with BMBC under surgery after neoadjuvant chemotherapy by SEER data, which could segment patients more precisely. We found that compared with chemotherapy alone, neoadjuvant chemotherapy plus surgical treatment significantly improved OS and BCSS in all molecular subtypes of patients with BMBC; however, this survival benefit depended on the metastatic burden. Only the patients with bone metastases only, bone and liver metastases, and bone and lung metastases could benefit from neoadjuvant chemotherapy plus surgical treatment, which indicated that the increase of metastatic burden, especially brain metastases reduced the effect of comprehensive treatment.

Despite the promising findings of the present study, there are some limitations of this research. First, although the SEER database covers about 30% of the USA population, clinical data on tumor subtypes and distant metastatic sites were collected only after 2010 in the SEER database and therefore limited the sample size of this study. Second, the SEER database offers a high representation of a general situation, but on the other side, not necessarily are suitable for applying to the Asian and Chinese populations on the basis of ethnic differences. Third, information about disease recurrence or subsequent sites of metastases was not collected in the SEER database. Thus, we could not investigate patients who developed bone metastases later in their remaining years, which may lead to bias in the results. Fourth, detailed treatment information for patients with bone metastases is not recorded in the SEER database, we cannot evaluate more on this. Even though the machine learning prognostic model achieved a higher accuracy rate, it lacked external validation to further enforce the reliability.

In summary, we constructed a machine learning prognostic model to provide a quantitative method to predict the survival of patients with BMBC, and our validation results indicate that this model should be highly reproducible in a similar patient population. We also identified potential prognostic factors for patients with BMBC and suggested that primary surgery followed by neoadjuvant chemotherapy therapy might increase survival in a selected subgroup of patients.

## Data availability statement

The original contributions presented in the study are included in the article/Supplementary material, further inquiries can be directed to the corresponding authors.

## Author contributions

Conceptualization: CL, JQ, and SZ. Methodology: CL and JQ. Formal analysis: CL. Data curation: ML. Writing—original draft preparation: JL, CF, and WW. Writing—review and editing: FW, XZ, CD, and YC. Supervision: YW and YZ. All authors have read and agreed to the published version of the manuscript.

## Funding

This work was funded in part by the following: National Science Foundation of China (81903856 to XZ; 82174164 to SZ; 82103569 to JQ); Key Science and Technology Program of Shaanxi Province (2021KW-57 to XZ; 2021KW-60 to JQ). Scientific research fund of the Second Affiliated Hospital of Xi'an Jiaotong University [RC(XM)202004 to XZ]. Free exploring fund of Xi'an Jiaotong University (xzy012022096 to XZ; xzy012022097 to JQ). Medical basic - clinical integration and innovation project of Xi'an Jiaotong University (YXJLRH2022088 to JQ).

## Conflict of interest

The authors declare that the research was conducted in the absence of any commercial or financial relationships that could be construed as a potential conflict of interest.

## Publisher's note

All claims expressed in this article are solely those of the authors and do not necessarily represent those of their affiliated organizations, or those of the publisher, the editors and the reviewers. Any product that may be evaluated in this article, or claim that may be made by its manufacturer, is not guaranteed or endorsed by the publisher.

## Supplementary material

Supplementary Figure 1 Confusion matrix of the XGBoost model's predicted results in the test set. **(A)** Confusion matrix in the 1-year prognostic model. **(B)** Confusion matrix in the 3-year prognostic model. **(C)** Confusion matrix in the 5-year prognostic model. TP, true positive; TN, true negative.

Supplementary Figure 2 The ranking of clinical characteristics in terms of importance in the XGBoost prognostic model. **(A)** The ranking of clinical characteristics in terms of importance in the 1-year prognostic model. **(B)** The ranking of clinical characteristics in terms of importance in the 3-year prognostic model. **(C)** The ranking of clinical characteristics in terms of importance in the 5-year prognostic model. XGBoost, extreme gradient boosting.

Supplementary Table 1 Results of the COX regression analysis [hazard ratio (HR), 95% CI, *p*-value].
