# Supplementary figures and images for: Machine learning predicts the prognosis of breast cancer patients with initial bone metastases
Source: Front Public Health. 2022 Sep 26;10:1003976. doi: 10.3389/fpubh.2022.1003976 (PMC9549149; doi:10.3389/fpubh.2022.1003976)

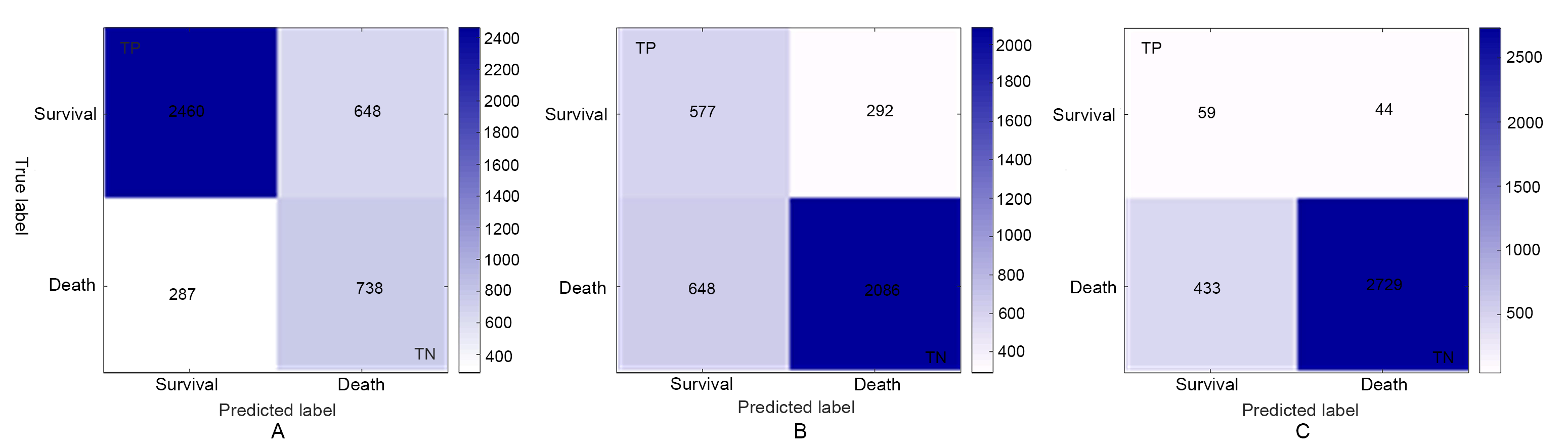

Supplement: Supplementary Figure 1 — Confusion matrix of the XGBoost model's predicted results in the test set. (A) Confusion matrix in the 1-year prognostic model. (B) Confusion matrix in the 3-year prognostic model. (C) Confusion matrix in the 5-year prognostic model. TP, true positive; TN, true negative. [file Image_1.TIF]

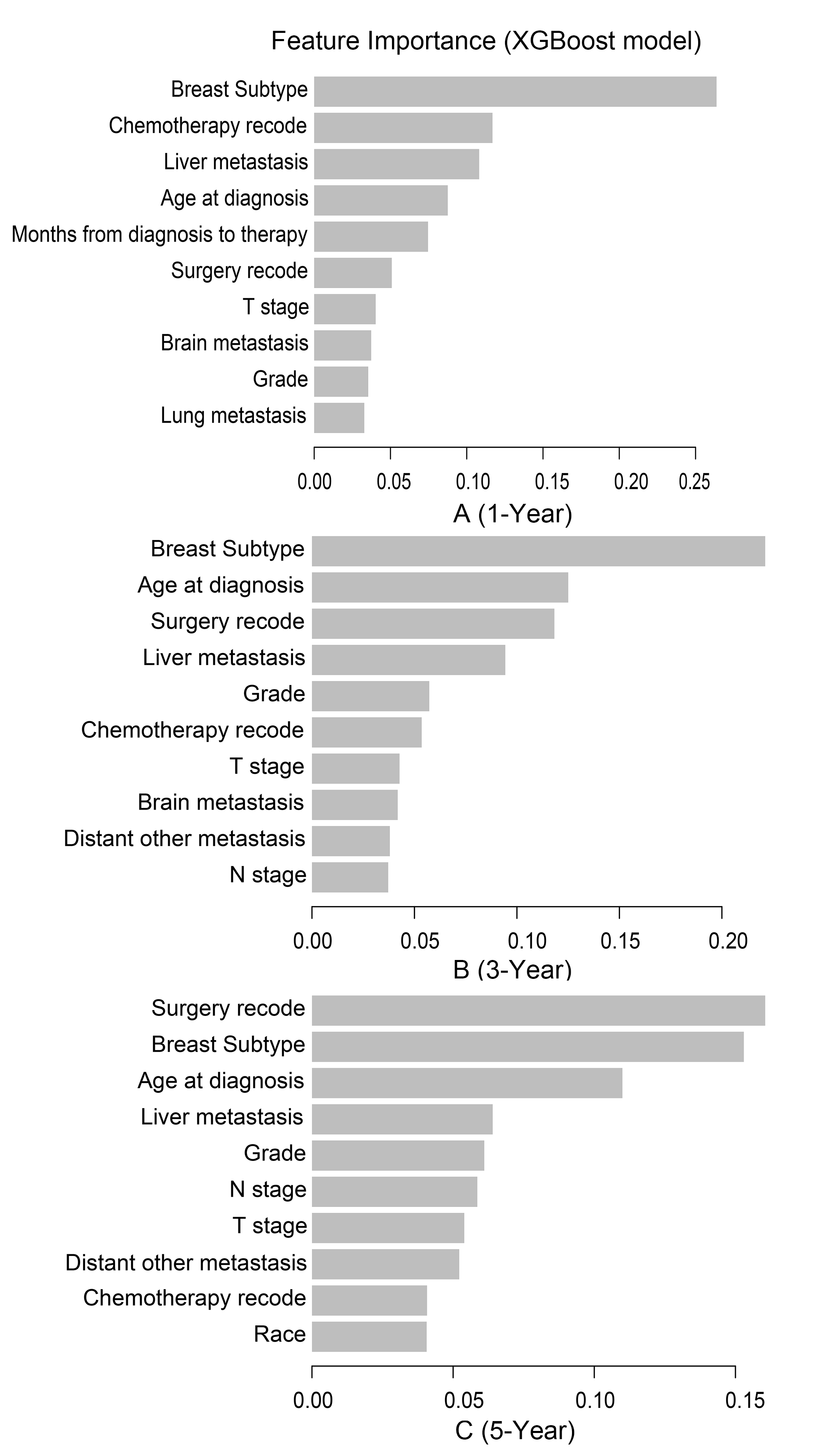

Supplement: Supplementary Figure 2 — The ranking of clinical characteristics in terms of importance in the XGBoost prognostic model. (A) The ranking of clinical characteristics in terms of importance in the 1-year prognostic model. (B) The ranking of clinical characteristics in terms of importance in the 3-year prognostic model. (C) The ranking of clinical characteristics in terms of importance in the 5-year prognostic model. XGBoost, extreme gradient boosting. [file Image_2.TIF]
